# Beyond the Neuron: TDP-43 and the Challenge of Pre-Analytical Quality in Platelet Biomarker Development

**DOI:** 10.3390/ijms27219691

**Published:** 2026-10-29

**Authors:** Birhanemaskal Malkamu, Emanuele Buratti, Sara Cappelli

**Affiliations:** 1 Department of Medical Laboratory Sciences, College of Health Sciences, Debre Tabor University, Debre Tabor P.O. Box 272, Ethiopia; birhanemlk@gmail.com; 2 Molecular Pathology Group, International Centre for Genetic Engineering and Biotechnology (ICGEB), 34149 Trieste, Italy; cappelli@icgeb.org

**Keywords:** TDP-43, biomarker, pre-analytical quality, platelets, ALS, FTD, proteomodulation

## Abstract

TAR DNA-binding protein 43 (TDP-43) is the primary pathological hallmark of amyotrophic lateral sclerosis (ALS) and frontotemporal dementia (FTD). Yet, despite two decades of intensive research, a reliable peripheral TDP-43 biomarker remains elusive. Recognizing platelets as a major blood reservoir of TDP-43 opens new diagnostic avenues but may also reveal potential vulnerabilities. In this narrative review, we first trace the evolving understanding of TDP-43 from a nuclear RNA-binding protein to a molecule whose functions and disease-associated alterations extend beyond the neuron. We then examine recent breakthroughs demonstrating that TDP-43 function is shaped by its cellular environment and how it may be uniquely concentrated in platelets. Next, we argue that pre-analytical variables, such as hemolysis, clotting, cold-chain failures, and biobank protocols, are not mere “noise” but potential confounders that distort molecular integrity and should be taken into account. Finally, we discuss the biological and methodological uncertainties surrounding platelet TDP-43 and highlight how standardized handling and systematic pre-analytical reporting can improve reproducibility and support future biomarker validation. More broadly, the methodological standards outlined in this review may extend beyond TDP-43 to other disease-related proteins that are present in platelets and similarly sensitive to pre-analytical variability.

## 1. Introduction

The transformation of a molecular hallmark into a clinical biomarker is rarely linear. For TAR DNA-binding protein 43 (TDP-43), translation of its well-established pathological relevance into a reliable peripheral biomarker has remained challenging. First discovered as a human immunodeficiency virus 1 (HIV-1) regulatory factor [1], TDP-43 was later recognized as a key RNA-binding protein involved in splicing regulation [2]. The paradigm shifted when TDP-43 was identified as the primary pathological protein in most cases of amyotrophic lateral sclerosis (ALS) and in approximately half of frontotemporal dementia (FTD) [3]. Despite this central role, the translation of TDP-43 into a reliable peripheral biomarker has not yet been established, principally because findings across biofluids, analytical methods, and clinical cohorts remain inconsistent [4].

This review focuses on platelet-derived TDP-43 and the pre-analytical factors that may influence its measurement in blood-based matrices, including isolated platelets, platelet-containing plasma, and nominally platelet-poor plasma. We specifically examine how pre-analytical variables may affect the reliability of platelet TDP-43 measurements and propose a reporting framework to improve comparability across future studies. In particular, we examine how inadequate control of pre-analytical conditions may contribute to this variability and discuss ways to address it. In general, it has been well recognized that pre-analytical variability is a general major source of error and irreproducibility in clinical laboratory medicine [5]. For TDP-43, this issue is particularly relevant because platelets constitute a TDP-43-enriched blood compartment [6]. Consequently, differences in platelet recovery, residual platelet content, activation state, and sample fractionation may influence measured TDP-43 concentrations. In addition, the biological complexity of platelets and the use of biobank protocols, that are very often designed without platelet-specific considerations, may introduce handling-dependent variables and complicate the distinction between biological signals and pre-analytical effects [6,7].

Moreover, we integrate two domains that are typically siloed in neurodegeneration research: molecular TDP-43 biology and clinical laboratory quality management. From the molecular perspective, we draw on recent evidence that TDP-43 is strongly influenced by the cellular environment. Indeed, its abundance, subcellular localization, RNA-binding targets, and effects on transcript processing may vary across cell types, indicating that observations derived from neuronal models cannot necessarily be extrapolated to peripheral tissues [8]. In addition, we integrate insights from clinical laboratory quality management to examine how time to centrifugation, storage temperature, hemolysis, clotting, and other pre-analytical variables affect sample quality and analyte integrity [5,9]. Despite its maturity in routine diagnostics, this knowledge has rarely been applied to neurodegenerative biomarker development.

Beyond their traditional role in hemostasis, platelets are increasingly recognized as multifaceted regulators involved in immune response, intercellular communication, and the sensing of systemic pathological processes [7,10,11]. Indeed, their granules, extracellular vesicles, surface receptors, and molecular cargo allow platelets to participate in neurovascular and inflammatory processes relevant to neurological disease [12,13]. More specifically, experimental studies indicate that activated platelets may contribute to selected neurodegeneration-associated processes, as described for Alzheimer’s disease (AD) and Huntington’s disease (HD). For example, in mouse models of AD, platelet-mediated beta-amyloid (Aβ) transport and accumulation have been associated with increased blood–brain barrier (BBB) permeability, cerebral Aβ deposition, and cognitive impairment, although the relevance of these findings to human disease remains uncertain [14,15]. Disease-associated platelet alterations have also been reported in HD, including changes in aspartate content and impaired angiogenic, thrombotic, and vascular functions in platelets enriched in mutant huntingtin [16,17]. In contrast, preliminary evidence from ALS studies suggests that platelets may carry measurable disease-associated signals. In ALS, platelet serotonin levels have been associated with survival, while platelet TDP-43 was reported to be elevated in sporadic disease, further supporting the potential of platelets as accessible sources of disease-associated molecular information [18,19]. These findings provide biological plausibility for platelet involvement in neurodegeneration, supporting the investigation of platelets as a potential source of disease-associated molecular information, while recognizing that biomarker validity remains to be established.

## 2. Beyond the Neuron: The Evolving Cellular and Peripheral Landscape of TDP-43

### 2.1. The Canonical View: A Nuclear RNA-Binding Protein in Neurodegeneration

TDP-43 is a ubiquitously expressed nuclear protein that regulates multiple aspects of RNA metabolism, including splicing, transport, stability, and translation [2,20]. Under physiological conditions, TDP-43 primarily resides in the nucleus, where it binds thousands of RNA targets and maintains cellular homeostasis [21,22]. However, under pathological conditions, such as ALS and FTD, TDP-43 mislocalizes to the cytoplasm, where it aggregates into insoluble inclusions. These cytoplasmic aggregates are now recognized as the defining pathological hallmark in approximately 97% of ALS cases, while TDP-43 pathology is estimated to underlie 45–50% of FTD cases [3,23,24].

The canonical view has therefore always been neuron-centric: TDP-43 pathology is considered a process primarily involving the central nervous system (CNS) [25], with peripheral manifestations receiving comparatively less attention [26,27]. This perspective has shaped biomarker development for nearly two decades, directing most efforts toward cerebrospinal fluid (CSF) and, to a lesser extent, plasma, both of which are assumed to reflect CNS pathology [28,29].

### 2.2. The Cellular Environment as a Primary Determinant of TDP-43 Function

Recent evidence indicates that the cellular environment, together with genetic variation and post-translational modifications, is an important determinant of TDP-43 function [8]. Using cell-type specific models, Šušnjar and colleagues systematically compared the transcriptomic and splicing consequences of TDP-43 depletion in cell lines of neuronal and skeletal muscle origin. The two cell lines had distinct baseline transcriptomes, which accounted for more variation than TDP-43 depletion in principal component analysis. Their results showed that the transcripts and alternative splicing events regulated by TDP-43 differed substantially between neuronal and muscle-derived cellular contexts, which may contribute to the observed differences in TDP-43-dependent regulation. TDP-43 depletion altered the splicing of canonical targets, such as polymerase (DNA-directed), delta interacting protein 3 (*Poldip3*) and sortilin 1 (*Sort1*), but only a limited proportion of transcriptomic and splicing changes was shared between the two cell types [8,30]. Beyond the shared targets, cell-type-specific splicing events and transcriptomic changes were observed, indicating that TDP-43 regulates distinct sets of transcripts depending on cellular context. The limited overlap persisted when analyses were restricted to genes expressed in both cell types, indicating that baseline transcript availability alone did not explain their different responses [8].

Taken together, these findings indicate that observations obtained in neuronal models cannot necessarily be extrapolated to other cellular contexts. Instead, TDP-43 appears to be a context-dependent regulator, whose function emerges from interactions with cell-specific mRNAs, RNA-binding proteins, and other components of the splicing machinery [2,22]. This context dependence provides a rationale for evaluating TDP-43 function in peripheral cellular compartments and exploring their potential as sources of TDP-43 biomarkers. However, the cited studies were conducted in nucleated cell lines; therefore, their results cannot be directly extrapolated to anucleate platelets, which lack nuclear transcription and have a distinct RNA regulatory environment.

### 2.3. Challenging the Neuron-Centric View: Evidence for Peripheral TDP-43 Biology

Over the past decade, accumulating evidence has forced a reevaluation of this neuron-centric paradigm. Although canonical TDP-43 proteinopathy remains mostly neuronal, detectable TDP-43 abnormalities have been reported in selected peripheral tissues and circulating cellular compartments [8,31,32].

*Skeletal muscle:* Evidence for TDP-43 pathology in skeletal muscle remains heterogeneous. Phosphorylated TDP-43 deposits have been detected in intramuscular nerve bundles and skeletal muscle fibers from patients with ALS [32,33]. Moreover, TDP-43 dysregulation has also been linked to metabolic abnormalities in the preclinical literature [34].

*Immune cells:* Peripheral blood mononuclear cells (PBMCs) from ALS patients exhibit disease-associated transcriptomic alterations. A recent study showed that combining the expression levels of *protein kinase cAMP-dependent type I regulatory subunit alpha* (*PRKAR1A*), *glutaminyl-peptide cyclotransferase* (*QPCT*), and *transmembrane protein 71* (*TMEM71*) distinguished ALS patients from healthy controls across two independent PBMC cohorts. Furthermore, knockdown of *PRKAR1A* reduced nuclear TDP-43 staining and increased the cytoplasmic accumulation of phosphorylated TDP-43 in motor neurons derived from induced pluripotent stem cells (iPSCs) from patients with ALS [35].

*Platelets:* Most significantly for biomarker development, platelets have emerged as a major peripheral reservoir of TDP-43. Recently, Luthi-Carter, Buratti and colleagues demonstrated that, under the fractionation conditions used in their study, at least 95% of the TDP-43 detected in platelet-containing plasma was localized within the platelet cytosol. Under these conditions, platelet-poor plasma contained less than 5% of the TDP-43 measured in platelet-rich plasma [6]. This “<5%” figure refers to the relative amount of TDP-43 in the two plasma fractions, not to the percentage of residual platelets. Together, these results have profound implications: blood-based TDP-43 measurements may be strongly influenced by residual platelet content unless platelet recovery and sample fractionation are adequately controlled.

Among the peripheral compartments discussed above, this review focuses on platelets because they are readily accessible through routine blood sampling and constitute a TDP-43-enriched compartment. These features make platelets particularly relevant for investigating how pre-analytical handling affects blood-based TDP-43 measurements. Preliminary studies have also reported disease-associated differences in platelet TDP-43. These findings are discussed in the following sections and motivate further evaluation of its biomarker potential, although evidence supporting its disease specificity and clinical value remains limited.

### 2.4. Clinical Relevance: Why Peripheral TDP-43 Biology Matters for Biomarker Development

Peripheral blood specimens may provide accessible matrices for TDP-43 biomarker development and disease monitoring, for two distinct reasons: the accessibility of blood sampling and the possibility of detecting disease-associated molecular signals. Blood can be collected repeatedly with minimal invasiveness, avoiding the need for lumbar puncture [4,36]. This practical advantage facilitates serial sampling but does not, by itself, establish the validity or clinical utility of a peripheral TDP-43 biomarker.

Platelets are particularly interesting because they are easily collected and contain abundant TDP-43 protein. Preliminary studies have reported disease-associated differences in platelet TDP-43 in ALS [37,38]. However, they do not establish that platelet TDP-43 is disease-specific or that its changes precede the onset of CNS symptoms.

Recent transcriptomic studies have demonstrated that platelets exhibit disease-associated molecular signals. For example, Arnaldo and colleagues performed RNA sequencing profiling of platelets from patients with idiopathic REM sleep behavior disorder (IRBD), dementia with Lewy bodies (DLB), Parkinson’s disease (PD), AD, and healthy controls. They observed extensive transcriptomic remodeling in IRBD and DLB, affecting RNA processing, cytoskeletal organization, and platelet activation pathways, while PD and AD showed minimal changes [39]. These results support the broader feasibility of platelet-based biomarker research, but they do not provide direct evidence about platelet TDP-43.

Turning to direct TDP-43 measurements, studies in CSF, plasma, and serum have yielded differing results.

For example, early studies reported an association between plasma phosphorylated TDP-43 and neuropathologically confirmed frontotemporal lobar degeneration with TDP-43 inclusions (FTLD-TDP), as well as increased total TDP-43 concentrations in the CSF of patients with ALS [40,41]. In contrast, An and colleagues developed and analytically validated a sensitive Meso Scale Discovery-based electrochemiluminescence immunoassay that detected lower full-length TDP-43 concentrations in plasma and serum from patients with ALS than in healthy controls [42]. More recently, biomarker development has expanded beyond direct protein quantification to include functional consequences of TDP-43 loss of function. In particular, cryptic exon-derived HDGF-like protein 2 (HDGFL2) peptides have been detected in CSF from symptomatic and presymptomatic *chromosome 9 open reading frame 72 (C9orf72) repeat expansion carriers*, providing a potential readout of impaired TDP-43-mediated splicing repression [43]. Even more recently, a new detection method based on the functional activity of TDP-43 measured using serum-based homogeneous time resolved FRET (hTR-FRET) showed that this was increased in ALS patients compared to controls [44].

These observations are not directly interchangeable: they concern different biological fluids, TDP-43 species or functional outcomes, and analytical methods. This variability underscores the need to define the analyte and standardize specimen collection and processing. Since platelet content can influence plasma TDP-43 measurements [6], this review focuses on platelet-derived TDP-43 and the pre-analytical factors that may affect its measurement.

## 3. Platelet TDP-43: Biological Rationale and Biomarker Potential

### 3.1. Platelet Biology and the Emerging Role of Platelets as Biomarker Sources

Platelets are anucleate cell fragments derived from megakaryocytes that range from 1 to 5 µm in diameter in humans. They contain functional mRNAs, microRNAs, and a diverse array of proteins distributed across the cytosol, membranes, organelles, and storage granules whose contents can be rapidly released upon activation [45,46,47]. Human platelets have a circulating lifespan of approximately 7–10 days [48,49]. In a murine model of immune-complex-mediated inflammation, activated platelets underwent transient sequestration, released their granular contents, and subsequently returned to the circulation. This observation suggests that activation and degranulation need not imply immediate platelet clearance, and raises the possibility that circulating platelet samples can include platelets with different activation histories. However, it does not establish whether or how recirculation affects TDP-43 abundance, processing, or release in human platelets. Its relevance to human TDP-43 measurement remains to be determined [50].

Platelet activation involves changes in shape and surface markers, protein synthesis from pre-existing mRNAs, and the release of granule contents [47,51]. These processes are relevant to specimen handling because activation can alter the molecular composition of the platelet sample. Activation and secretion responses also depend in part on the agonist and signaling pathway involved, as illustrated by differences between platelet releasates generated by collagen and thrombin stimulation [52]. Through these properties, platelets can communicate with multiple cell types and influence vascular, inflammatory, and immune processes.

Although historically appreciated for their role in hemostasis and thrombosis, platelets have recently emerged as a promising source of biomarkers for multiple pathological conditions. Platelet transcriptomic and proteomic profiles have been associated with cardiovascular disease and platelet reactivity [53,54,55,56]. Platelets have also been associated with cancer and infection [57,58]. Reported findings include associations of thrombocytosis with cancer outcomes [57,59,60,61], associations of thrombocytopenia with severe infection outcomes [62], and interactions between platelets and microbial components [58,63].

Finally, the molecular content of platelets offers a promising and readily accessible window into disease-associated changes in neurological disorders [6,19]. Indeed, functional similarities between platelet and neuronal cells, including secretion mechanisms [64,65] and vesicular compartmentalization [64,66], provide a biological rationale for investigating platelet molecular changes in neurological disease. Platelet alterations have been reported in PD [67] and multiple sclerosis [68], while platelet expression and release of cellular prion protein provide a connection with prion-related processes [69]. Platelet-associated changes have also been described in HD and AD [17,70]. These observations support investigating platelets in TDP-43 proteinopathies such as ALS, but do not establish the specificity or clinical validity of platelet TDP-43 measurements.

Importantly, for biomarker development, three distinct readouts must be distinguished: platelet TDP-43 protein, transcripts of the TAR DNA-binding protein gene (*TARDBP*) encoding TDP-43, and downstream RNA signatures potentially associated with TDP-43 function. TDP-43 protein measurements assess the protein itself; *TARDBP* transcript measurements assess its encoding RNA and cannot be assumed to reflect protein abundance or processing. Downstream RNA signatures concern other transcripts or RNA-processing events and require separate evidence linking them to TDP-43 activity in platelets. In particular, detecting transcripts previously identified as TDP-43 targets in other cell types does not establish the same regulatory activity in platelets. Evidence supporting platelets as a source of disease-related information in ALS includes both indirect molecular associations and direct TDP-43 protein measurements.

Even before platelet TDP-43 was investigated directly, platelet-associated alterations had been linked to clinically relevant disease features. Specifically, Dupuis et al. [18] reported that platelet serotonin levels were associated with survival in patients with ALS, indicating that platelet molecular content may provide information related to disease progression independently of direct TDP-43 measurement. More direct evidence was subsequently provided by Hishizawa et al. [19], who compared platelets from patients with sporadic ALS with those from healthy controls and reported significantly higher platelet TDP-43 levels in the ALS group. This study provided an initial proof of concept that disease-associated differences in TDP-43 can be detected directly within the platelet compartment, although validation in larger and independently characterized cohorts remains necessary.

The interpretation of these findings also requires consideration of platelet biology. The platelet transcriptome is not replenished through nuclear de novo transcription because platelets lack a nucleus. Thus, platelet RNA signatures may provide a molecular record shaped by megakaryocyte-derived transcripts, platelet age, and interactions with the circulating environment. However, their interpretation requires control of platelet purity, activation state, processing time, and RNA integrity [71,72]. Conversely, platelet protein content is highly dynamic, shaped by ongoing granule release, activation-induced secretion, protein synthesis, rapid degradation and proteolytic processing [47,73].

Protein-based measurements, such as TDP-43 protein levels, may therefore be particularly sensitive to pre-analytical variables including collection-induced platelet activation, temperature fluctuations, centrifugation, and storage duration. Nonetheless, RNA- and protein-based readouts should both be considered susceptible to pre-analytical variation and require endpoint-specific validation [6,71,74].

These considerations have direct methodological implications for TDP-43 measurement in blood. When platelets are the intended specimen, their purity and activation state require control; when nominally cell-free plasma is analyzed, platelet carryover may contribute to the measured signal. Platelets may therefore represent either the intended source of TDP-43-related information or an uncontrolled contributor to plasma measurements, depending on specimen preparation and analytical design [6,7].

### 3.2. Platelets as a Major but Pre-Analytically Vulnerable Blood Reservoir of TDP-43

As previously discussed, the study by Luthi-Carter and colleagues identified platelets as a major blood compartment for TDP-43 and further characterized the biological and methodological implications of this localization for biomarker development [6]. Using fractionated human blood, they demonstrated that most of the TDP-43 protein detected in platelet-rich plasma derived from platelet cytosol. Platelet RNA sequencing additionally detected transcripts of the *TARDBP* gene, along with numerous transcripts previously identified as targets of TDP-43 regulation in neuron-like and astrocyte-like cellular models. Furthermore, the authors showed that standard plasma preparation protocols, including centrifugation steps and platelet carryover near the buffy coat, can strongly affect apparent plasma TDP-43 levels [6]. Therefore, elevated TDP-43 in plasma may reflect variable platelet contamination or recovery rather than a stable disease-associated signal. This is biologically plausible because platelets are anucleate, megakaryocyte-derived fragments that retain a complex repertoire of proteins, RNAs, granules, and extracellular-vesicle-associated cargo [10,75,76,77]. The retention of these components may provide a biological basis for considering residual platelets as a potential source of signal in plasma assays.

Beyond protein and RNA abundance, disease-associated changes have also been identified in the platelet transcriptome. Arnaldo and colleagues [39] reported differences in platelet RNA composition across diagnostic groups, including a lower proportion of long non-coding RNAs (lncRNAs) in DLB. These findings support investigating platelet RNA signatures, but do not establish a relationship with platelet TDP-43 abundance or activity. Their relevance to TDP-43 biomarker development therefore depends on demonstrating such a relationship rather than inferring it from disease-associated transcriptomic differences alone.

At a mechanistic level, platelets may influence the brain through the release of bioactive molecules and extracellular vesicles (EVs), contributing to the modulation of neuroinflammation, neuroprotection, and neuroregenerative processes [78]. For biomarker measurement, the relevant consideration is that platelet-derived EVs can carry inflammatory mediators and disease-associated proteins [10,69], making platelet origin and activation potential contributors to the composition of blood EV preparations. Experimental detection of platelet-derived microvesicles in the mouse hippocampus provides context for possible peripheral-to-brain cargo transfer [79], but does not establish transfer of platelet TDP-43 or validate blood EV TDP-43 as a measure of brain pathology. Accordingly, EV-associated TDP-43 measurements require assessment of residual platelets, platelet-derived EV contributions, and activation during specimen processing.

However, the same biological responsiveness that makes platelets attractive as a source of candidate biomarkers also makes them vulnerable to pre-analytical variability. Specifically, platelets are metabolically active and sensitive to mechanical and thermal stress, as well as centrifugation, and delays in sample processing [6,74]. These findings have profound implications, because routine blood collection, storage, or biobanking can activate or destabilize platelets, potentially altering the distribution of their cargo, including TDP-43, microRNAs, and other candidate biomarkers.

The implication is that TDP-43 measurements may be strongly influenced by platelet recovery, residual platelet content, or activation, highlighting the need for appropriate pre-analytical controls during sample collection and processing. Notably, most published biobank protocols do not consistently document platelet activation status and residual platelet content, rendering historical datasets difficult to interpret [6].

## 4. Pre-Analytical Stressors as Structured Confounders in Platelet-TDP-43 Biomarker Analysis

The vulnerability of platelets to activation may contribute to pre-analytical variation. The pre-analytical phase, which encompasses sample collection, transport, processing and storage, is recognized as the most error-prone segment of the testing pathway, and may account for up to 70% of laboratory errors [5].

**Figure 1:**
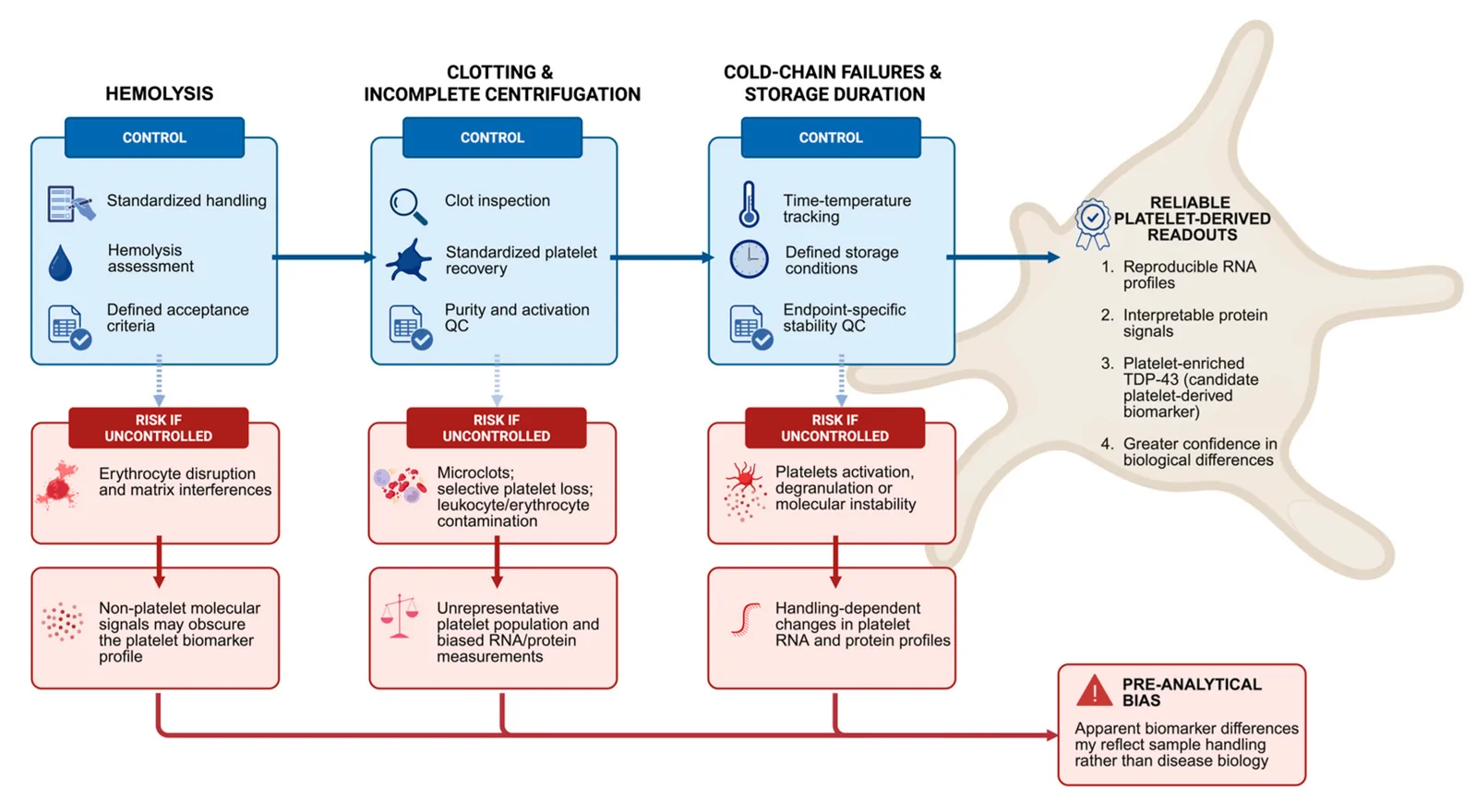
Pre-analytical control framework for platelet-derived TDP-43 biomarker studies. Hemolysis is a common pre-analytical problem [9] and may introduce erythrocyte-derived interference and alter assay performance [80]. Clotting, centrifugation, anticoagulant choice, and cellular carryover can alter platelet recovery and sample purity [6,71,81,82,83]. Temperature, storage duration, and freeze–thaw cycles may affect platelet activation and molecular stability [42,74,84]. Standardized, endpoint-specific handling supports reliable platelet-derived readouts and reduces pre-analytical bias [5,71,85,86]. Created in BioRender. Cappelli, S. (2026) https://BioRender.com/6l7a1l0 (accessed on 28 September 2026).

Regarding platelet-derived TDP-43 measurements, three key pre-analytical variables should be carefully assessed, as they can potentially affect platelet recovery, sample composition, and analyte stability: (1) hemolysis, (2) clotting/incomplete centrifugation, and (3) cold-chain failures and storage duration. These groups address potential assay interference, platelet recovery and cellular carryover, and specimen stability, respectively. Their relative importance for platelet-derived TDP-43 measurements remains to be established. Figure 1 presents this pre-analytical control framework. Table 1 summarizes the supporting evidence, specifying the specimen matrix in which each factor has been studied, whether TDP-43 was measured directly, the observed effects, and the remaining hypotheses that require further investigation. Together, they distinguish potential sources of measurement variability from demonstrated effects on TDP-43 and identify where direct validation is still required.

### 4.1. Hemolysis

The rupture of erythrocytes, known as hemolysis, introduces hemoglobin, proteases, and oxidative species into the sample. Malkamu and colleagues [9] documented hemolysis rates as high as 10–15% in multicenter hospital settings in Ethiopia. Interestingly, hemolysis can interfere with antibody-based measurement [80] and alter circulating RNA profiles [87], representing a potential source of bias in blood-based biomarker development.

Most importantly, the severity of hemolysis is not binary. In 2025, Thorn and colleagues quantified hemolysis using the hemolysis index (H index), with a score of 1 corresponding to a hemoglobin concentration of 11–130 mg/dL. Their experiments using hemolyzed serum revealed that slight hemolysis (H index = 1) did not interfere with accurate quantification of most cytokines examined, suggesting that low-grade hemolysis may be acceptable for certain analytes [80]. However, the effects of hemolysis varied according to the analyte and analytical platform. It is important to note that the cited cytokine and RNA findings should not be read as defining a TDP-43 tolerance for hemolysis.

Critically, hemolysis and platelet activation may arise from the same collection-related conditions. For instance, a patient with difficult venous access may experience both erythrocyte rupture and platelet activation during the same blood collection attempt. Despite this, hemolysis indices and platelet activation status are not consistently documented, rendering archived samples difficult to interpret and reflecting broader concerns about pre-analytical reporting [5].

For TDP-43 protein measurements, hemolysis represents a potential confounding variable because it may interfere with immunoassay performance. However, its specific influence on TDP-43 measurements requires systematic characterization. Hemolysis assessment should therefore be incorporated into TDP-43 biomarker studies. The findings discussed here should be regarded as biologically plausible extrapolation rather than demonstrated analytical effects.

### 4.2. Clotting and Incomplete Centrifugation

Clot formation and non-standardized centrifugation can alter platelet recovery and sample composition. For nominally cell-free plasma, the relevant concern is the contribution of residual platelets to measured TDP-43 levels. Variations in centrifugation speed and duration can cause different amounts of platelets to sediment, resulting in variable residual platelet content across samples. In studies of domestic and food-producing animals, higher centrifugal forces generally remove more platelets from plasma, whereas lower forces may leave more platelets in suspension [81]. In human samples, platelet recovery and carryover have been shown to influence measured plasma TDP-43 concentrations [6]. Clot formation can trap platelets within fibrin networks, potentially leading to variable recovery of platelet-associated TDP-43. This potential effect is distinct from centrifugation-related variability and should be documented separately. However, the effect of clot formation on TDP-43 recovery has not been directly quantified in these studies.

Recent evidence quantifies the magnitude of the centrifugation effect. In a study comparing two plasma isolation methods across 50 breast cancer patients, direct centrifugation (DC) yielded a median residual platelet count of 1.0 × 10^9^/L, while density gradient centrifugation (DGC) resulted in 3.5 × 10^9^/L, corresponding to a 3.5-fold increase in residual platelet content [82]. This difference cannot be interpreted as an equivalent change in TDP-43 concentrations. Although this study did not investigate TDP-43 directly, its findings provide context for those of Luthi-Carter and collaborators, who showed that plasma TDP-43 levels are strongly influenced by platelet recovery and platelet carryover [6]. Therefore, for platelet-enriched biomarkers such as TDP-43, differences in residual platelet content may substantially affect the apparent plasma signal and represent an important source of variability across studies.

The choice of anticoagulant further complicates matters, particularly depending on the downstream readout. For plasma RNA, peptide, and vesicle-associated measurements, a recent study found that both anticoagulant type and plasma-processing conditions can influence platelet-derived signals, including miRNAs, peptides, microparticles, and vesicle-associated fractions [86]. For EV-focused analyses, ethylenediaminetetraacetic acid (EDTA) plasma has been shown to enrich specific extracellular vesicle (EV) subpopulations, including platelet-associated EVs, compared with acid-citrate-dextrose (ACD)-plasma [88]. For platelet function and proteomic measurements, a recent study showed that EDTA increases platelet α-granule secretion, whereas other anticoagulants, including sodium citrate and ACD, produce distinct patterns of interference [89]. Therefore, anticoagulant selection should be validated according to the platelet-derived endpoint being investigated. However, many TDP-43-related studies do not report anticoagulant type, making cross-study comparison challenging. Direct evidence for the effect of anticoagulant choice on platelet TDP-43 measurements is currently lacking, and the findings discussed here should be regarded as hypothesis-generating.

Although recent multiomic recommendations emphasize explicit documentation of centrifugation protocols (e.g., speed, duration, temperature, and brake settings) and acknowledge that EV-focused analyses may require dedicated processing workflows [86], many TDP-43-related studies have used different sample matrices and processing procedures, making cross-study comparisons challenging [4,6]. These observations highlight the need to standardize protocols.

For isolated platelet transcriptomics, an example of a defined processing workflow comes from Arnaldo and colleagues [39], who used a two-step centrifugation procedure: an initial centrifugation at 200× *g* for 15 min to obtain platelet-rich plasma, followed by a second centrifugation at 2500× *g* for 15 min to obtain platelet pellets. Importantly, all samples were processed within four hours of blood collection to limit expression changes linked to platelet activation. This workflow was used for platelet RNA profiling and does not establish optimal conditions for TDP-43 protein recovery or stability. However, it is consistent with some of the timeframes recommended by the French Society of Thrombosis and Haemostasis (SFTH) for hemostasis testing [84], although these recommendations were not developed specifically for platelet transcriptomics. More broadly, standardized processing is considered essential for the reliability of platelet RNA sequencing data [71].

Recent studies have further characterized the magnitude of pre-analytical bias in platelet-containing samples. Bead-based proteomics approaches, while enhancing the detection of low-abundance proteins, may be susceptible to systematic bias arising from platelet and PBMC carryover [83]. Investigations have demonstrated that centrifugation conditions, anticoagulant choice, and buffer-bead combinations can influence contamination profiles, although these biases may be mitigated through optimized sample handling [83]. These observations concern proteomic contamination profiles and do not quantify a TDP-43-specific effect. For platelet flow cytometry, guidelines emphasize that pre-analytical variables must be carefully controlled to obtain biologically valid and robust results [90]. Taken together, these data reinforce the central argument of this review that rigorous pre-analytical standardization is not optional but essential for reliable platelet-based biomarker studies.

### 4.3. Cold-Chain Failures and Storage Duration

Platelet activation is temperature-dependent and time-dependent. Prolonged storage at either 4 °C or room temperature can modify platelet activation and degranulation responses [74]. Temperature fluctuations, processing delays, and repeated freeze–thaw cycles may also disrupt platelet membranes or alter the distribution of intracellular contents. These variables are particularly relevant to platelet-associated analytes because they may affect platelet integrity and the distribution of molecular cargo between cellular and extracellular compartments.

#### 4.3.1. Evidence from Platelet Concentrates

Winskel-Wood and colleagues [74] demonstrated that cold-stored platelets retained their capacity to respond to stimuli after 21 days of storage but exhibited a progressive increase in procoagulant platelet formation (reaching ~20% by day 21). Although these outcomes cannot be directly extrapolated from platelet concentrates to biobank samples, they demonstrate that storage conditions can progressively alter platelet phenotype and responsiveness. Such changes could influence the distribution and measurement of platelet-derived TDP-43, although their specific effects and kinetics remain insufficiently characterized.

#### 4.3.2. Evidence from Plasma and Serum

An et al. [42] directly evaluated the stability of TDP-43 in plasma and serum during repeated freeze–thaw cycles using an electrochemiluminescence immunoassay. The first two to three cycles had minimal effects on measured concentrations, whereas a modest reduction was observed after the fourth cycle in plasma and after the third or fourth cycle in serum. These data indicate that repeated freeze–thawing may introduce additional variability into TDP-43 measurements and that freeze–thaw history should be documented. However, these results concern TDP-43 measured in plasma and serum and do not establish its stability in isolated platelet preparations, which requires separate validation.

#### 4.3.3. Implications for Specimen Handling and Reporting

Recommendations from SFTH emphasize that acceptable storage and processing intervals depend on the analyte, sample matrix, temperature, and intended assay [84]. Although these recommendations were developed primarily for hemostasis testing rather than platelet transcriptomics or TDP-43 measurements, they reinforce the importance of documenting time to processing, storage temperature, and deviations from validated conditions.

Analytical platform and biological matrix must also be considered. Sensitive immunoassays have enabled the quantification of full-length TDP-43 in serum and plasma, while electrochemical platforms have demonstrated the technical feasibility of detecting low TDP-43 concentrations in serum [42,91]. Nevertheless, differences in assay design, sample matrix, platelet content, and storage history may influence the measured signal and complicate comparisons across studies.

To improve reproducibility, TDP-43 biomarker studies should therefore adopt structured sample documentation, such as the Standard PREanalytical Code (SPREC) or a comparable reporting framework. SPREC records key biospecimen variables and should be supplemented with freeze–thaw history, storage duration, and other assay-specific handling information [85]. Applying such frameworks to platelet-derived TDP-43 studies would facilitate the identification of potentially compromised samples, improve comparability across cohorts, and support quality-based interpretation of molecular measurements.

Overall, Table 2 summarizes the main sources of variability discussed above and their potential implications for blood-based TDP-43 measurements.

## 5. Pre-Analytical Validation and Reporting of Platelet TDP-43: Current Gaps and Future Directions

Despite significant advances in understanding TDP-43 molecular biology and its presence across different cellular and peripheral compartments, translating this knowledge into a clinically actionable peripheral biomarker remains obstructed by several critical gaps. These gaps span basic platelet biology, sample stability, and reporting standards. Below, we systematically outline each gap, its clinical relevance, and proposed research directions. Future studies should determine whether handling-induced variation is large enough to obscure or mimic a disease-associated difference.

### 5.1. Defining the Pre-Analytical Stability of Platelet TDP-43 Protein and RNA Readouts

This gap directly relates to the unresolved question from Section 4.3 concerning whether storage-induced changes in platelet phenotype and cargo distribution measurably affect platelet TDP-43 concentrations. Although platelet activation and degranulation change progressively during storage, the time- and temperature-dependent stability of platelet TDP-43 has not been systematically characterized. Studies of other platelet-associated molecules, including serotonin, CD62P/P-selectin, and platelet factor 4 (PF4), demonstrate that platelet phenotype and cargo release are influenced by storage conditions and temperature [74,92,93]. Whether platelet TDP-43 protein, its proteoforms, and *TARDBP* RNA follow comparable kinetics remains unknown. Stability should be evaluated separately for total TDP-43, full-length and phosphorylated proteoforms, and RNA-based readouts (including both TARDBP transcripts and downstream TDP-43-responsive RNAs).

This gap prevents the definition of acceptable processing delays and storage conditions for platelet-derived TDP-43 measurements. Samples processed at different time points may yield different TDP-43 concentrations because of changes in platelet activation, integrity, or cargo distribution rather than disease-related biology. Although full-length TDP-43 appears relatively stable during the first freeze–thaw cycles in plasma and serum, these findings cannot be directly extrapolated to isolated platelet preparations or other TDP-43 proteoforms [42].

Future studies should therefore examine platelet-associated and extracellular TDP-43 simultaneously across controlled combinations of processing time, temperature, storage duration, and freeze–thaw exposure. Stability should be evaluated separately for total TDP-43, specific proteoforms (including full-length and phosphorylated forms), and RNA-based readouts. Established laboratory-medicine frameworks can then guide the design and reporting of these experiments [5,84].

### 5.2. Disease-Associated Variation in Platelet TDP-43 and Pre-Analytical Susceptibility

Building on the unresolved effects of anticoagulants, centrifugation, and platelet carryover discussed in Section 4.2, this subsection asks whether these handling effects differ between patients and controls. Platelets from patients with neurodegenerative diseases may differ biologically from those of healthy controls. Disease-associated changes in amyloid precursor protein processing, platelet activation and reactivity, and transcriptomic profiles have been described in AD and synucleinopathies [39,94,95,96]. In ALS, preliminary studies have reported alterations in platelet TDP-43 abundance and proteoform distribution, but these observations remain limited and require independent replication [6,19].

It remains unknown whether disease-associated platelets differ in their susceptibility to activation, membrane disruption, or cargo release during sample processing. If platelets from patients and controls respond differently to identical handling conditions, measured differences in TDP-43 could reflect a combination of biological variation and differential pre-analytical sensitivity. This possibility cannot be resolved by standardized handling alone and requires direct assessment of platelet activation and integrity in each diagnostic group.

Arnaldo et al. [39] provided evidence that platelet transcriptomic profiles vary across neurodegenerative conditions. Extensive differential expression was observed in idiopathic REM sleep behavior disorder and dementia with Lewy bodies, whereas fewer changes were detected in PD and AD. These findings demonstrate that platelet molecular profiles may be disease-dependent, but they do not establish altered platelet fragility or directly characterize TDP-43 biology.

Future case–control studies should therefore combine platelet TDP-43 measurements with platelet counts, recovery estimates, activation markers, cellular-purity controls, and disease-relevant molecular profiling. Controlled ex vivo perturbation experiments could determine whether ALS- or FTD-associated platelets respond differently to delays, temperature changes, centrifugation, or freeze–thaw exposure. Such studies are necessary to distinguish authentic disease-associated TDP-43 alterations from differences introduced or amplified during sample handling. Using paired aliquots from the same blood draw, these experiments should quantify handling-induced changes within each diagnostic group and compare their magnitude with the patient–control difference observed under a common reference condition, to determine whether handling can obscure, amplify, or mimic a disease-associated signal.

### 5.3. Lack of Standardized Pre-Analytical Reporting in TDP-43 Biomarker Studies

This issue directly relates to the unresolved question from Section 4 concerning whether the lack of standardized reporting prevents meaningful cross-study comparison of platelet TDP-43 measurements. Available TDP-43 biomarker studies show substantial heterogeneity in the reporting of pre-analytical procedures [4,28]. Information on the interval between blood collection and processing, centrifugation conditions, anticoagulant type, hemolysis, clotting, and residual platelet content is not consistently provided. This lack of standardized documentation limits cross-study comparisons and may complicate future quantitative evidence synthesis.

In clinical laboratory medicine, established guidelines describe the collection and handling variables that should be controlled and documented [97]. For example, the Clinical and Laboratory Standards Institute guideline H21-A5 provides recommendations for the collection, transport, and processing of blood specimens for coagulation testing, including centrifugation and storage conditions [98]. However, no comparable reporting guideline has been developed specifically for blood-based or platelet-derived TDP-43 biomarker studies.

Based on established pre-analytical recommendations and laboratory-medicine frameworks [5,97,99], we propose the minimum reporting checklist for blood-based and platelet-derived TDP-43 studies presented in Table 3.

## 6. Method

This narrative review synthesizes literature identified through PubMed, Google Scholar, and Web of Science.

The last search was conducted in August 2026. Principal search strings included: (“TDP-43” OR “TARDBP”) AND (“platelet” OR “thrombocyte”); (“TDP-43” OR “TARDBP”) AND (“biomarker” OR “biomarker development”); (“pre-analytical” OR “preanalytical”) AND (“platelet” OR “blood”); (“hemolysis” OR “haemolysis”) AND (“biomarker” OR “assay”); (“centrifugation” OR “platelet recovery”) AND (“plasma” OR “serum”); (“storage stability” OR “freeze-thaw”) AND (“platelet” OR “TDP-43”); (“ALS” OR “amyotrophic lateral sclerosis”) AND (“platelet” OR “blood biomarker”); and (“FTD” OR “frontotemporal dementia”) AND (“platelet” OR “blood biomarker”).

Studies were prioritized according to four criteria: (1) they addressed platelet biology, TDP-43 biology, or pre-analytical quality; (2) they were peer-reviewed primary research articles, systematic reviews, or authoritative guidelines; (3) they were published within the last ten years, while foundational older studies were included for historical context; and (4) they directly measured TDP-43 in the relevant specimen matrix, with studies inferring effects from other analytes treated as indirect evidence.

Because this is a narrative review, we did not apply formal quality scoring or meta-analytic methods; instead, we focused on conceptual integration and identification of knowledge gaps. Nevertheless, we aimed for comprehensive coverage of the main pre-analytical variables affecting platelet-rich biofluids.

## 7. Limitations of the Review

This review has several important limitations. First, the evidence base for platelet TDP-43 remains limited, and much of the available evidence is indirect, derived from other analytes, specimen types, or experimental systems. Second, direct stability data for platelet TDP-43 under different pre-analytical conditions are scarce, and the specific effects of hemolysis, clotting, centrifugation, and storage on platelet TDP-43 have not been systematically characterized. Third, the current evidence does not allow ranking of pre-analytical factors by effect size, and their relative importance for platelet TDP-43 measurements remains to be established. These limitations should be considered when interpreting the findings and recommendations of this review. Finally, the selection of cited studies reflects the authors’ expert judgment. Accordingly, strong causal claims should be interpreted as hypotheses requiring further empirical validation.

## 8. Conclusions

A reliable peripheral biomarker of TDP-43 pathology is still lacking across TDP-43-associated neurodegenerative diseases. Recent evidence indicates that platelets are a readily accessible and TDP-43-rich blood compartment. Preliminary studies in ALS have also reported disease-associated differences in platelet TDP-43, supporting further investigation of platelets as a potential source of peripheral biomarkers [6,19]. However, these observations require independent replication, longitudinal assessment, and rigorous analytical validation.

In this review, we have brought together platelet TDP-43 biology and clinical laboratory quality management to examine how pre-analytical conditions influence the reliability of platelet-derived TDP-43 measurements. It is important to distinguish the observed influence of platelet recovery on apparent plasma TDP-43 from proposed effects of other handling factors on platelet TDP-43 abundance or integrity. Hemolysis, clotting, centrifugation conditions, temperature changes, storage duration, and uncontrolled platelet activation should not be regarded simply as technical noise. These factors can influence platelet recovery, activation state, molecular integrity, and cargo distribution, thereby altering measured TDP-43 levels. Blood-based TDP-43 results should consequently be interpreted with caution when relevant information about sample collection, processing, and storage is unavailable [5,6,97,98,99].

Future studies should use standardized procedures specifically designed for the analytical endpoint under investigation and clearly report the sample matrix, anticoagulant, processing time, centrifugation conditions, storage history, hemolysis, cellular purity, platelet recovery, and activation status.

Translating platelet TDP-43 from a promising research finding into a clinically useful biomarker will require both biological validation and reliable laboratory procedures. Combining sensitive analytical methods with standardized sample handling and transparent reporting will help establish whether platelet TDP-43 can provide reproducible diagnostic, prognostic, or treatment-related information, first in ALS and, if supported by future evidence, across the broader spectrum of TDP-43-associated diseases.

Overall, although the considerations discussed in this review were developed in the context of platelet-derived TDP-43, they are likely to be relevant to the broader development of platelet-based biomarkers. The same pre-analytical factors that may affect TDP-43 measurements, including sample handling, platelet recovery, activation status, cellular purity, and storage conditions, may also influence the measurement of other platelet-associated molecules. Accordingly, the framework proposed here may provide a useful basis for the design, standardization, and interpretation of future studies investigating platelets as a source of biomarkers across different diseases.

## Figures and Tables

**Table 1:** Summary of evidence on pre-analytical factors potentially affecting platelet-derived TDP-43 measurements, including the specimen matrices studied, whether TDP-43 was measured directly, observed effects, and unresolved questions.

Pre-Analytical Factor	Specimen Matrix	TDP-43 Measured Directly?	Observed Effect	Remaining Hypothesis
Hemolysis	Human and nonhuman serum for cytokine immunoassays [80]; human plasma for miRNA measurements [87]	Not directly measured in the cited studies [80,87]	Hemolysis can interfere with antibody-based assays [80] and alter RNA profiles [87]. These findings do not quantify an effect on TDP-43	The magnitude and direction of any effect on TDP-43 assay performance or platelet-associated RNA readouts remain to be established
Clotting, centrifugation, and platelet carryover	Plasma and platelet-rich plasma; platelet preparations obtained using different isolation methods	Yes, for plasma TDP-43 in platelet-carryover studies [6]. The platelet-content comparison [82] does not itself establish an effect on TDP-43	Platelet content differed between isolation methods [82]. Platelet carryover affected measured plasma TDP-43 [6]. Changes in platelet content cannot be assumed to produce proportional changes in TDP-43	The separate effects of clotting and centrifugation on TDP-43 recovery and distribution between platelet-associated and extracellular fractions remain to be quantified. Effects on isolated platelet TDP-43 require validation
Temperature, storage duration, and freeze–thaw cycles	Platelet concentrates in cold-storage studies; plasma and serum in freeze–thaw experiments	Yes, in plasma/serum TDP-43 stability experiments; not established for isolated platelet TDP-43	Repeated freeze–thaw cycles were reported to reduce full-length TDP-43 after 3–4 cycles [42]. Cold storage altered platelet phenotype [74], which does not itself demonstrate a change in TDP-43	Effects on isolated platelet TDP-43 abundance, processing, and release remain unquantified. Findings from plasma or serum cannot be assumed to apply to platelet preparations

Note: This table synthesizes evidence from platelet concentrate storage studies, plasma/serum TDP-43 stability experiments, and general laboratory medicine literature. Findings from these different specimen types cannot be directly extrapolated to isolated platelet preparations. The specific effects of these pre-analytical factors on platelet-derived TDP-43 therefore require endpoint-specific validation.

**Table 2:** Summary of storage-related evidence from platelet concentrates and plasma/serum TDP-43 experiments, with implications for isolated platelet measurements.

Pre-Analytical Factor	Main Concern for TDP-43 Measurement	Supporting Evidence	Implication for TDP-43
Room temperature (RT) storage	Progressive changes in platelet activation and degranulation; bacterial-growth risk during prolonged storage	Platelet activation and degranulation responses change during storage of platelet concentrates [74]	Processing time and temperature should be documented; the effects on TDP-43 require direct validation
Cold storage (4 °C)	Development of a storage-associated procoagulant phenotype	Cold-stored platelet concentrates retained responsiveness but showed progressive procoagulant platelet formation, reaching approximately 20% by day 21 [74]	Cold storage may alter platelet state and consequently influence platelet-derived measurements; its effect on TDP-43 stability remains unknown
Repeated freeze–thaw cycles	Membrane damage and redistribution or degradation of molecular contents	Full-length TDP-43 concentrations in plasma and serum were minimally affected by the first two to three cycles but modestly reduced after additional cycles [42]	Freeze–thaw history should be documented, and repeated cycles should be minimized through sample aliquoting

Note: This table summarizes evidence from platelet-concentrate storage studies and TDP-43 stability experiments in plasma and serum. Neither evidence source directly establishes TDP-43 stability in isolated platelet preparations. Storage effects therefore require specimen- and endpoint-specific validation.

**Table 3:** Proposed minimum pre-analytical reporting checklist for blood-based and platelet derived TDP-43 biomarker studies. Values in the “Example” column are provided for illustration only and do not represent validated acceptance thresholds or exclusion criteria.

Variable	Required Detail	Example
Sample matrix	Whole blood, serum, plasma type, isolated platelets, platelet lysate, or cytosolic fraction	Isolated platelet pellet
Phlebotomy-to-processing time	Minutes or hours	120 min
Anticoagulant type	EDTA, citrate, heparin, ACD, or none	ACD (acid-citrate-dextrose)
Centrifugation protocol	Speed (× *g*), duration (min), temperature (°C), and brake setting (yes/no)	1500× *g*, 10 min, 22 °C, brake off
Storage conditions (pre-centrifugation)	Recorded temperature (°C) and exact duration	22 °C for 120 min
Storage conditions (post-centrifugation)	Temperature (°C), duration, and number of freeze–thaw cycles	−80 °C for 6 months; one cycle
Hemolysis assessment	Quantitative or semiquantitative result, assessment method, and predefined acceptance criterion	Hemoglobin: 0.25 g/L; spectrophotometric method
Clot presence	Presence or absence of clots and predefined handling or exclusion criteria	No visible clot; samples with visible clots excluded
Platelet count and recovery	Measured counts in original whole blood and final plasma or platelet preparation, as applicable	Counts reported for each relevant fraction
Cellular purity	Residual leukocyte and erythrocyte content and assessment method	Residual leukocytes assessed by flow cytometry
Platelet activation status	Activation marker, analytical method, and measured value, where available	CD62P measured by flow cytometry
Sample quality decision	Included, excluded, or flagged, with reason	Included according to predefined criteria

## Data Availability

No new data were created or analyzed in this study. Data sharing is not applicable to this article.

## Abbreviations

The following abbreviations are used in this manuscript:

Aβ	Amyloid Beta
ACD	Acid-Citrate-Dextrose
AD	Alzheimer’s Disease
ALS	Amyotrophic Lateral Sclerosis
BBB	Blood–Brain Barrier
C9ORF72	Chromosome 9 Open Reading Frame 72
CD62P	P-Selectin
CNS	Central Nervous System
CSF	Cerebrospinal Fluid
DC	Direct Centrifugation
DGC	Density Gradient Centrifugation
DLB	Dementia with Lewy Bodies
EDTA	Ethylenediaminetetraacetic Acid
EVs	Extracellular Vesicles
FTD	Frontotemporal Dementia
FTLD-TDP	Frontotemporal Lobar Degeneration with TDP-43 Pathology
H index	Hemolysis Index
HD	Huntington’s disease
HDGFL2	HDGF-Like Protein 2
HIV-1	Human Immunodeficiency Virus Type 1
hTR-FRET	Homogeneous Time-Resolved Förster Resonance Energy Transfer
iPSCs	Induced Pluripotent Stem Cells
IRBD	Idiopathic REM Sleep Behavior Disorder
lncRNAs	Long Non-Coding RNAs
miRNAs	MicroRNAs
mRNAs	Messenger RNAs
PBMCs	Peripheral Blood Mononuclear Cells
PD	Parkinson’s Disease
PF4	Platelet Factor 4
Poldip3	Polymerase (DNA-Directed), Delta Interacting Protein 3
PRKAR1A	Protein Kinase cAMP-Dependent Type I Regulatory Subunit Alpha
QPCT	Glutaminyl-Peptide Cyclotransferase
REM	Rapid Eye Movement
RNA	Ribonucleic Acid
RT	Room Temperature
SFTH	French Society of Thrombosis and Haemostasis
Sort1	Sortilin 1
SPREC	Standard PREanalytical Code
TARDBP	TAR DNA-Binding Protein
TDP-43	TAR DNA-Binding Protein 43
TMEM71	Transmembrane Protein 71
